# Evaluating a complex model designed to increase access to high quality primary mental health care for under-served groups: a multi-method study

**DOI:** 10.1186/s12913-016-1298-5

**Published:** 2016-02-17

**Authors:** Christopher Dowrick, Peter Bower, Carolyn Chew-Graham, Karina Lovell, Suzanne Edwards, Jonathan Lamb, Katie Bristow, Mark Gabbay, Heather Burroughs, Susan Beatty, Waquas Waheed, Mark Hann, Linda Gask

**Affiliations:** Institute of Psychology, Health and Society, Waterhouse Building, University of Liverpool, Liverpool, L69 3GL UK; NIHR School for Primary Care Research, Manchester Academic Health Science Centre, University of Manchester, Manchester, M13 9PL UK; Primary Care and Health Sciences Research Institute, Keele University, Keele, Staffordshire ST5 5BG UK; School of Nursing, Midwifery and Social Work, Jean MacFarlane Building, University of Manchester, Manchester, M13 9PL UK; College of Medicine, Grove Building, Swansea University, Swansea, SA2 8PP UK; West Midlands Collaboration for Leadership in Applied Health Research & Care, Birmingham, UK

**Keywords:** Access, Quality, Under-served, Mental health, Multi-level intervention, Community, Primary care, Wellbeing

## Abstract

**Background:**

Many people with mental distress are disadvantaged because care is not available or does not address their needs. In order to increase access to high quality primary mental health care for under-served groups, we created a model of care with three discrete elements: community engagement, primary care training and tailored wellbeing interventions. We have previously demonstrated the individual impact of each element of the model. Here we assess the effectiveness of the combined model in increasing access to and improving the quality of primary mental health care. We test the assumptions that access to the wellbeing interventions is increased by the presence of community engagement and primary care training; and that quality of primary mental health care is increased by the presence of community engagement and the wellbeing interventions.

**Methods:**

We implemented the model in four under-served localities in North-West England, focusing on older people and minority ethnic populations. Using a quasi-experimental design with no-intervention comparators, we gathered a combination of quantitative and qualitative information. Quantitative information, including referral and recruitment rates for the wellbeing interventions, and practice referrals to mental health services, was analysed descriptively. Qualitative information derived from interview and focus group responses to topic guides from more than 110 participants. Framework analysis was used to generate findings from the qualitative data.

**Results:**

Access to the wellbeing interventions was associated with the presence of the community engagement and the primary care training elements. Referrals to the wellbeing interventions were associated with community engagement, while recruitment was associated with primary care training. Qualitative data suggested that the mechanisms underlying these associations were increased awareness and sense of agency. The quality of primary mental health care was enhanced by information gained from our community mapping activities, and by the offer of access to the wellbeing interventions. There were variable benefits from health practitioner participation in community consultative groups. We also found that participation in the wellbeing interventions led to increased community engagement.

**Conclusions:**

We explored the interactions between elements of a multilevel intervention and identified important associations and underlying mechanisms. Further research is needed to test the generalisability of the model.

**Trial registration:**

Current Controlled Trials, reference ISRCTN68572159. Registered 25 February 2013.

## Background

Mental health problems impose substantial emotional, social and economic burdens on those who experience them, their families and carers, and society as a whole [1]. A range of interventions and initiatives has been shown to be effective in clinical trials in improving outcomes for people experiencing common mental health problems [2, 3]. However many people with high levels of mental distress are disadvantaged because the benefits of these effective models are limited by problems in access: people may not be aware of or express a mental health need, or be aware of the availability of suitable services [4]. Care may not be available to them in the right place and time, or when they do access care their interaction with professional care-givers may deter help-seeking, or divert it to forms that do not address their needs [5, 6]. People from black and minority ethnic communities often have inadequate access to primary care. For example, women of South Asian family origin in the UK have a high prevalence of depression and self-harm, often in the context of severe and persistent social difficulties, which only become apparent when they are in a crisis [7]. Older people often receive inadequate help when they do access primary care. For example depression is common in older people, particularly those with chronic physical illness, but tends to be under-diagnosed and inadequately managed [8, 9].

Developing interventions to improve access to mental health care is a policy priority in many health care systems. Current policy initiatives tend to focus on supply-side factors [10–13]. There is less consideration of demand issues and factors influencing the journey of the person in need, including the community and social contexts within which mental health problems arise [14, 15].

### The AMP programme

**Fig. 1:**
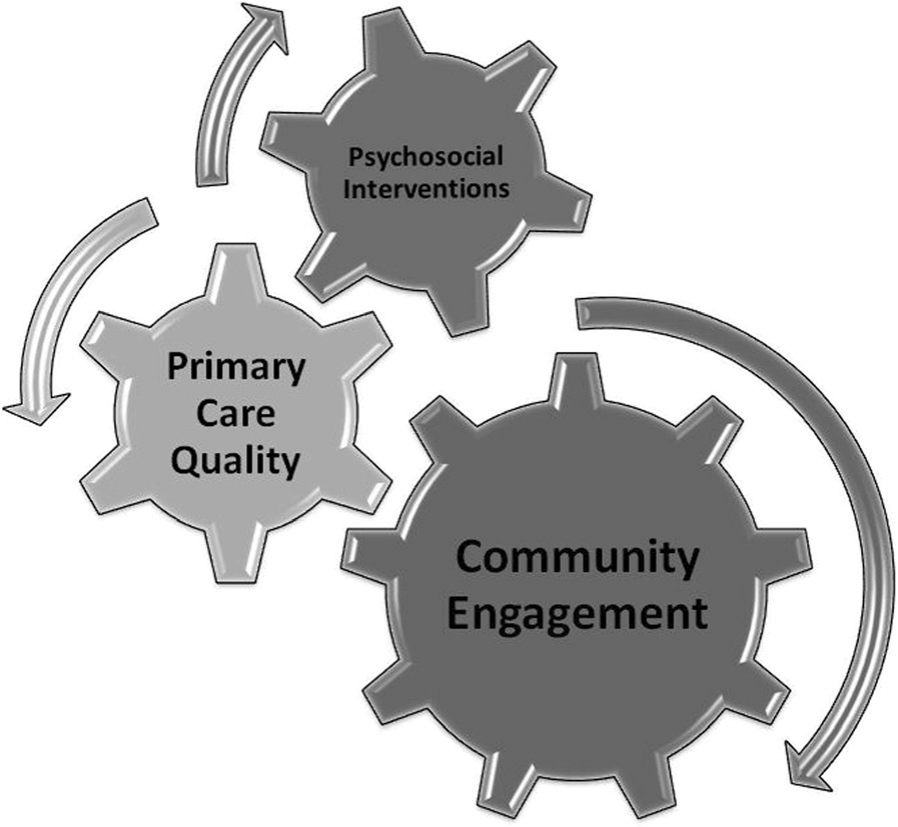
AMP development partnership

We undertook a research and development programme to improve access to high quality primary care mental health for people from under-served groups [16]. We began by clarifying the mental health needs of people from seven under-served groups, identifying relevant evidence-based services and barriers and facilitators for access to such services [4, 17–20]. On the basis of our findings, we designed and developed a multi-faceted intervention model with three elements: community engagement, primary care quality and tailored psychosocial interventions [21]. This model, which we called AMP (Improving **A**ccess to **M**ental Health in **P**rimary Care) is presented schematically in Fig. 1.

### Implementing the AMP model

We implemented the AMP model in four disadvantaged localities in Liverpool and Manchester, in North-West England. Each locality had a population in the region of 25,000, considered as the optimum size for delivering the full range of integrated primary care services [16], and contained wards (electoral subdivisions) rated within the most deprived 5 % in England [22]. We focused on older people in two localities and minority ethnic groups (South Asian or Somali) in the other two. These groups were chosen in line with the priorities of the local primary care organisations. In each locality we identified four main primary care teams (practices) to work with.

Our *community engagement* element had four components. Information gathering involved entry into the field, key informant interviews and mapping and collation of existing resources. ‘Community champions’ were identified to provide an interface between the research team and the interests of the local community. Community focus groups were created to negotiate the aims and agenda of the intervention with local people, agencies and wider stakeholders, and agree an action plan. A community working group was then set up to implement the action plan.

The *primary care* element composed an interactive training package, AMP training*plus,* which had three components. *Knowledge transfer:* we offered face-to-face training for up to six sessions, initially chosen from a menu of subject options. The training element began with a standard session, and then developed according to needs of particular practices. Topics included cultural understandings of mental health and health care, legal problems for asylum seekers and linking with local resources. *Systems review:* we undertook intensive observation centred on reception and appointment systems to identify organisational and structural features that may impede or promote access by underserved groups. *Active linking*: we raised awareness of other relevant organisations and resources mapped and logged by the AMP team.

For the *wellbeing intervention* element, we synthesised data from previous programme work streams and local focus group findings to design an intervention based on cognitive-behavioural principles, with an emphasis on social participation. Wellbeing facilitators (with experience in cognitive behavioural therapy, counselling or psychological wellbeing) were trained to deliver a patient-centred assessment leading to a choice of three pathways: individual sessions (up to eight), group sessions (eight to 10) or sign-posting to other relevant services. The mode and site of delivery depended on patient preference.

### Evaluation of each AMP model component

We have previously evaluated separately each of the elements of the AMP model [23–25].

More than 50 people took part in the community engagement element, including people from third sector organisations, housing associations, faith leaders, police, local councillors, business leaders, health practitioners and commissioners. There was strongest engagement with third sector organisations (more than 20 participants), and variable engagement with health practitioners and commissioners. Outputs included innovative ways to improve health literacy, including calendars and relaxation CDs. A case study focusing on the South Asian community project found that it provided opportunities to share experiences, rebuild links between third sector organisations and develop links between these organisations and primary care. Establishing a focused agenda enabled local communities to raise the agenda of mental health and wellbeing amongst many other priorities in an area of multiple deprivation [23].

We offered AMP training*plus* to eight practices, of which seven agreed to participate. Practices varied in the extent to which team members other than GPs were involved in the training. There was also variation in the number of training sessions completed, with a range from one to seven [24]. Staff who engaged with the training programme reported increased awareness, recognition and respect for the needs of patients from under-served communities. Changes in style and content of interactions were reported, particularly amongst receptionists, and there was evidence of system change. The training program also increased awareness of and encouraged signposting to local community agencies. Our qualitative evaluation indicated that practice engagement was facilitated by prior knowledge of the research team, the presence of a champion within the practice, and a sense of co-production of the training [24].

We tested the feasibility and acceptability of the wellbeing intervention for ethnic minority and older people in an exploratory randomised trial. Over 15 months we recruited 57 patients (57 % of target) with high levels of unmet need, mainly through GPs. While recruitment was less than expected, our qualitative data showed that patients found the content and delivery of the intervention acceptable. The individual wellbeing sessions were the most popular mode of delivery. Quantitative analysis indicated that the 37 patients randomised to receive the wellbeing interventions improved compared to those receiving usual care [25].

### Objectives of this paper

In this paper we explore the interactions between the three elements of the AMP. Specifically, we test two assumptions:

1. that access to wellbeing interventions is increased by the presence of the community engagement and primary care training elements; and
2. that quality of primary mental health care is increased by the presence of the community engagement and wellbeing intervention elements.

For the first assumption, we specify access in terms of referral (initial expression of interest in the wellbeing interventions) and recruitment (fulfilling criteria for the wellbeing interventions). We would expect access to the wellbeing interventions to be greater in the two localities which were offered the full AMP community engagement programme, and for patients registered with one of the seven practices which participated in AMP training*plus;* and greatest for those who were involved with both the community engagement and the practice training elements.

For the second assumption, we would expect the presence of the other two elements to stimulate the expansion of perspectives and activity amongst participating practices beyond conventional healthcare parameters, specifically showing evidence of engagement with third-sector organisations and the under-served community.

## Methods of evaluating the integrated AMP model

**Fig. 2:**
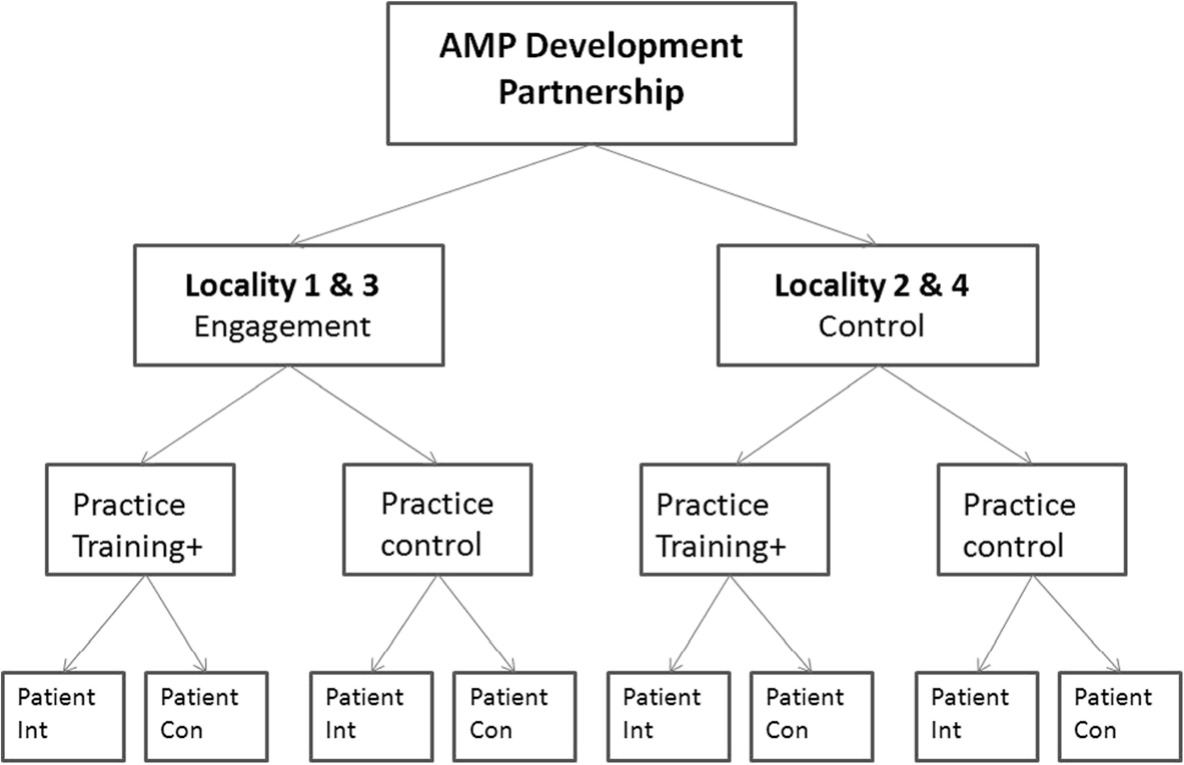
Schema of evaluation process. Int = Intervention; Con = Control

An important challenge was to develop ways of evaluating the integrated model, capturing both process and outcome and enabling any interactions between components to be identified and assessed. We therefore undertook a multi-level evaluation to test the assumption that intervening at three levels would be mutually reinforcing and more effective than intervening at one or two levels [26]. We used a quasi-experimental design with a no-intervention comparator for each element (see Fig. 2).

We randomised the four localities so that two received the full community engagement element, while two acted as controls and only participated in information gathering. We offered the full community engagement package to the South Asian community in one Manchester locality (Locality 3) and the older white community in one Liverpool locality (Locality 1). The Somali community in the other Liverpool locality (Locality 2) and older white community in the other Manchester locality (Locality 4) acted as controls. As noted above, residents in all four localities experienced similar high levels of deprivation. In Locality 2 more than 33 % residents identified as non-white, while in Locality 3 more than 50 % residents identified as non-white [16].

Within each locality we identified the four main practices and randomised them so that two were offered AMP training*plus,* while the other two acted as controls. The eight practices offered the intervention had a mean list size of 6865 patients (range 2743 to 11,710). The eight control practices had a mean list size of 6213 patients (range 2570 to 9247).

For the wellbeing intervention, we randomised at the individual patient level [25].

In order to explore the interaction of the elements of the model, and their relative importance, we gathered project-specific and routine quantitative data. To assess the impact of the other two elements of the model on access to the wellbeing intervention, we gathered data on the numbers of patients referred and recruited to the intervention, and related this to the locality and to the practice. Referral of potential participants could be from GPs, other health professionals, the voluntary sector or self-referral. Recruitment of patients into the wellbeing intervention was then dependent on consent and fulfilment of entry criteria specified by the research team [25]. To assess the impact of the primary care training on the community engagement element, we tracked referrals to mental health and wellbeing services in both intervention and control practices, using standardised primary care electronic record codes.

In order to understand the processes involved in increasing access to the wellbeing interventions, and to gather perspectives on the impact of the integrated model on the quality of primary mental health care, we collected qualitative data from more than 110 informants through purposively sampled interviews and focus groups. We conducted 19 interviews with people who had participated in our consultative focus groups in the community engagement localities and with an equivalent range of respondents in the control localities; we also undertook three focus groups with local community mental health and wellbeing organisations. Six of the seven practices which took part in AMP training*plus* agreed to provide follow-up information: we conducted 13 individual interviews and undertook two focus groups with six and eight participants respectively. We interviewed 39 patients who had participated in the wellbeing intervention, as well as all the wellbeing facilitators and two of their supervisors: the third supervisor provided reflective notes.

The content of the interviews and focus groups was informed by detailed topic guides. For community engagement interviewees we included prompts about factors influencing relations between voluntary agencies and primary care and whether they had heard of the wellbeing intervention. For primary care interviewees we included questions about whether they had experience of the wellbeing interventions and (for respondents in community engagement localities) whether the community engagement element had impacted on their practice. For wellbeing intervention interviewees we explored perceived barriers and enablers to the wellbeing intervention, with prompts about promoting access to primary mental health care.

We used a combination of quantitative and qualitative methods to analyse our data, our focus being appropriateness to research and evaluation questions rather than a formally mixed-methods design [27, 28]. Our quantitative analyses were descriptive, as sample sizes were insufficiently powered for formal comparative analyses. We subjected qualitative data to Framework analysis [29, 30], an approach suited to an environment where multiple researchers are gathering information from differing sites (reported in detail [23]). We developed an initial coding framework from themes grounded in the data and concepts identified in the review phase and checked transcripts against it to ensure no significant omissions. Using the qualitative software package MAXQDA [31], we further synthesized codes first across individual transcripts and iteratively across the entire data set, before reorienting this to the a priori evaluation framework. Work with primary data drew on Constant comparative analysis [32] and the synthesis of themes in Meta-Ethnography [33] to generate broader categories and linked codes across interviews [23]. We interpreted and analysed data within this evaluative framework to distil, structure and judge statements about the impact of the combined interventions.

Ethical approval for the community engagement and primary care elements of the study was granted by Northwest 6 Research Ethics Committee, reference 09/H1004/67. Ethical approval for the wellbeing intervention was granted by North West 8 Research Ethics Committee, reference 10/H1003/38. The wellbeing intervention was registered under current controlled trials, reference ISRCTN68572159. Participants in the community engagement and primary care elements provided verbal consent, participants in the wellbeing intervention provided written consent.

## Results

### 1. Access to psychosocial interventions

**Fig. 3:**
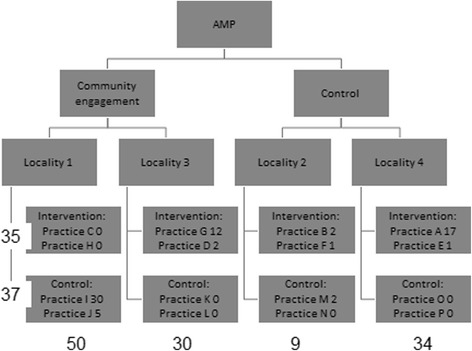
Referrals to AMP wellbeing intervention, by locality and practice. Numbers in rows are of practice referrals. Numbers in columns are of locality referrals (including referrals from practices and other sources)

Referrals to the wellbeing interventions were much more likely to come from the two localities which experienced AMP community engagement. Figure 3 shows that there were 80 referrals from all sources in these localities, compared with 43 from all sources in the other two localities. Fifty one (41 %) referrals were from community organisations and self-referrals. Referral was not associated with the offer of AMP training*plus*. There was no difference in the combined number of referrals from primary care teams offered (*n* = 35) or not offered (*n* = 37) the AMP primary care intervention. Thirty referrals came from one control practice.

**Fig. 4:**
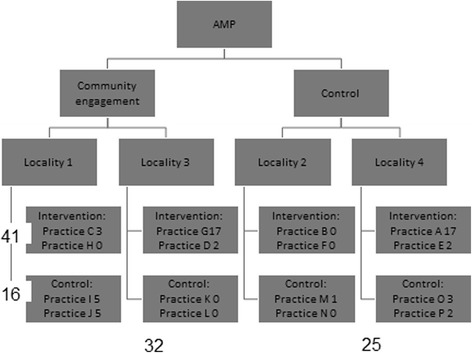
Recruitment to AMP wellbeing interventions, by locality and practice. Both column and row numbers are of patients registered with practices

Recruitment to (i.e. fulfilling criteria for) the wellbeing interventions, conversely, was associated with the offer of AMP training*plus*, but not with community engagement (see Fig. 4). Forty one (72 %) of the 57 participants recruited to the wellbeing intervention were registered with a practice offered AMP trainingplus, while only 16 (18 %) were registered with a practice not offered the training. Thirty two (56 %) of recruited participants came from one of the two localities which had received the community engagement intervention, while 25 (44 %) came from one of the other two localities*.* Recruited participants were more likely to be referred by GPs in intervention practices (27/57, 42 %) than by any other source, Control GP referrals led to 9 (15 %), community organisations to 17 (30 %) and self-referrals to 4 (7 %) recruits.

**Fig. 5:**
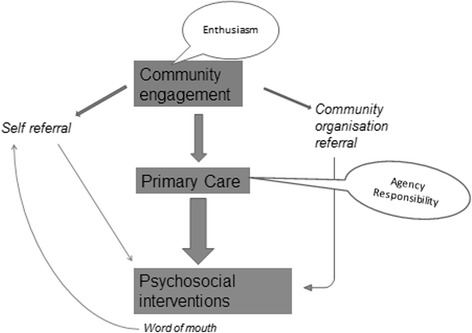
Schema of referral patterns

Our qualitative findings help to explain the mechanisms underlying these patterns (see Fig. 5). We focus here on the themes of community awareness and professional agency. The community engagement element of the model enhanced awareness of the wellbeing intervention, especially amongst voluntary groups, and hence encouraged referrals. Levels of acceptability were similar amongst community groups and in primary care. However it was a sense of agency amongst GPs, coupled with the knowledge gained from participation in AMP training events, which was associated with successful recruitment into the wellbeing intervention.

*Awareness* of the AMP wellbeing intervention was more influenced by the community engagement element than by the practice training element. All of the 16 AMP practices were invited to refer patients to the wellbeing interventions, regardless of whether they were invited to participate in training. The absence of a community engagement intervention in Locality 2, combined with the fact that many members of the Somali community gather information by word-of-mouth rather than through written materials, contributed to low levels of awareness about the psychosocial intervention in that locality.

The wellbeing interventions were welcomed within practices and local communities, and by service commissioners:

*"I think the great thing that you're doing is you're trying to catch them before they go into that severe depression stage, and work on them mild to moderate before they're at that severe stage[.…]. And I work with other organisations with cognitive behavioural therapy and it's about making them aware of how they're feeling and changing that into a positive. So I think it's a great idea…"* Housing trust community worker, Locality 4

*“So the AMP is improving access to mental health and primary care and then you have got the wellbeing facilitators … and … really exciting and well done for the bit that says in our, you know, here are the … facilities, here are the local things that we have in our local community … which for us in a practical world is really helpful. Really helpful for GPs, really good to say, listen here’s, you know, here’s what you’ve got.”* Service commissioner, Localities 1 &2.

There was a stronger sense of *agency* amongst primary care teams than amongst community organisations or potential self-referrers. GPs are used to taking responsibility and making decisions about whether and when to refer to other care providers. Family doctors who had been offered AMP training*plus* were in a strong position to enable effective recruitment (see Fig. 4), as their sense of agency was combined with greater awareness of the patients most likely to benefit.

Members of community organisations, in contrast, were less likely to feel responsible or powerful enough to make referrals. Although good will and empathy were evident, they often did not perceive themselves as having the authority or the appropriate structures within which to act:

“*I think it could be that as well that they [workers at voluntary organisation] don’t feel comfortable referring directly and doing it through a GP somehow takes the onus off them.*” Community worker, Locality 4.

*“If there was anyone that I was sort of concerned about, I always discuss it with the vicar. And, you know, if there’s anything we can do in that way then by all means, but I wouldn’t be making decisions by myself; I don’t think it would be part of my role.”* Project worker, Locality 1.

### 2. Primary care quality

The quality of the primary care element of the AMP model, in the sense of awareness and activity in relation to third sector organisations and under-served communities, was influenced by both the community engagement and the wellbeing intervention elements.

#### Community engagement

Within the community engagement element, we found evidence of effects of community mapping and the consultative and community working groups.

The *community mapping* activity was seen as useful by practices engaged with AMP training*plus*. Several teams chose identifying locality resources and services for mental health support as the focus of their training sessions, expressing needs for accessible, updated information on current service provision. AMP research staff who carried out the community mapping attended selected training sessions. Benefits were reported by practices in terms of providing them with new information on valuable local community resources:

*“They were very useful. It made the practice team aware of existence of groups and services that we never thought existed around us……Also it gave us a clear way of tapping into these services whenever we need them, so that is a great benefit.”* Family doctor, Locality 2.

These observations are supported by quantitative data. Analysis of 18 months of electronic health records of six practices offered with AMP training*plus* showed a total of 23 referrals to voluntary services. This contrasted with zero referrals to voluntary services during the same period from three of the practices which were not offered AMP training*plus.*

There were two differing patterns of interaction between primary care teams and the *consultative and working group* components of the community engagement element.

In Locality 3, the involvement of primary care team members in initial community meetings encouraged their later participation in AMP training*plus*, overcoming previous hesitation or reluctance*.* It also encouraged the development of relationships with voluntary organisations:

*“Yeah the community ones they were really good, very good and … that was really really positive … to have the GPs all there together. The government wants us to move towards working a lot more closely with the GPs so it’s an excellent thing to have in our community*.” Family service worker, Locality 3

In Locality 1, in contrast, the community meetings were initially attended mainly by stakeholders from the voluntary sector. Our active ‘selling’ of the wellbeing intervention to both training and control practices encouraged the participation of family doctors and other community health professionals in later community meetings. However it is unclear how far this led to productive dialogue:

*“Primary care were at one side of the table and the voluntary, I was talking with the voluntary sector we were at the other side.”* Health Commissioner, Locality 1.

Thus it appears that early engagement of practice members with local community meetings was more likely to lead to productive dialogue with voluntary organisations and to expand primary care perspectives outside conventional healthcare boundaries.

#### Wellbeing intervention

The offer of the wellbeing intervention was a means of initial and ongoing engagement with primary care teams. As with locality mapping, it positioned AMP as offering a service to the teams, and hence increased the likelihood of their participation in the training elements of AMP training*plus*.

However, we found three ways in which implementation of the wellbeing intervention at practice level could be problematic. One obstacle was that practice staff could not be certain that referred individuals would be eligible for or offered the service. Second, the wellbeing intervention was not embedded in everyday practice of family doctors: some saw it as requiring extra time in an already busy environment, hence easiIy forgotten:

“*That 10 minute consultation you know, they’ll refer to whatever is at the forefront of their minds so maybe a more of a plug for the service and a, you know, a constant reminder about it*.“ Practice Manager, Locality 3.

Third, some doctors found difficulty in identifying suitable candidates for the wellbeing service, especially from within specific ethnic minority groups:

*“I think the problem is because it’s just a specific group of patients…Although we have got Somali patients, they are not, we have also got a massive group of other patients from ethnic minorities… I didn’t realize it was going to be specifically for the Somali population…And that probably narrowed it really’.* Family doctor, Locality 2.

This led to a recommendation for a more inclusive approach.

### Wellbeing and community engagement

Although we were not formally investigating the impact of the AMP wellbeing intervention on the community engagement element, we were presented with evidence of a positive feedback loop in this direction.

Some patients who had experienced the AMP wellbeing interventions felt sufficiently empowered by their experience to offer their time to work with local community organisations, specifically to raise awareness about mental health issues:

*“I can work as a volunteer, I know what depression is what people go through and what can help to recover really have that experience after so many years. The AMP group and the [community] group are really good ones and I would suggest people to join them.”* Pakistani woman, Locality 3.

“*I can work as a role model for people and can voluntarily come to your group sessions and tell the participants how I overcame my depression naturally with the help of you people and you have made me strong.”* Pakistani woman, Locality 3.

## Discussion

### Summary of findings

Our integrated model, with its simultaneous community, primary care and psychosocial interventions, and its layered experimental design, is unique in the existing academic literature. The use of a quasi-experimental design in evaluation provides opportunities for assessing components and their interactions more clearly than would be possible in a simpler implementation design. The small number of quasi-experimental ‘units’ (localities and practices) puts significant limits on the quantitative yield, but combining allocation of model components with qualitative methods does provide scope for linking quantitative outcomes to a detailed analysis of the processes that might underlie them. This approach enabled us to explore elements of this complex intervention more effectively than a more traditional experimental design could.

Acknowledging caveats around the precision of the quantitative data, we have demonstrated the added value of this approach in relation to its effects on access to psychosocial interventions and on the quality of primary mental health care. Referrals to the wellbeing intervention were associated with community engagement, while recruitment was associated with the offer of AMP training*plus;* our qualitative analysis indicates mechanisms concerning enthusiasm amongst community groups and a greater sense of agency amongst family doctors. With regard to the impact of the other components of the AMP model on primary care quality, our community mapping activities were seen as strongly positive; there were varying benefits from interaction between practice training and participation in community groups; and the offer of the wellbeing intervention enhanced engagement with practice training.

Our initial focus was on the potential multiplicative effects of the multi-level model. Our qualitative data also highlighted the importance of feedback loops, which might only be picked up qualitatively over time.

### Limitations

The inherent complexity of the evaluation process rendered data collection and analysis challenging. We needed to exercise judgment when drawing inferences across and between differing methodological perspectives, and to remain aware of the requirement to draw pragmatic conclusions of relevance to health care planners and policy makers.

There was a tension between achieving the core aims of the AMP programme and maintaining sufficient flexibility to engage and maintain effective partnerships with our research stakeholders. Our evaluations were focused more on process than on outcomes, although understanding process is a crucial aspect of identifying effective interventions [34]. Our findings regarding outcomes were descriptive: our sample sizes were not sufficient to merit the application of conventional statistical techniques to estimate significance, nor to directly address questions about equity of access. The locality where our South Asian community lived was different in a number of ways from the community of Somalis included in this research, including relative population density and duration of residence. It was not possible for researchers conducting interviews to be blind to the position of informants within the AMP programme.

When considering how the different elements of the AMP model affected each other, we do not imply that these were fixed entities. They represented a dynamic set of processes. The elements did not routinely have set or rigid boundaries, but were defined by ongoing negotiation of boundaries between and within the three elements.

### Implications for research and practice

Further analysis is needed to elucidate the impact of the AMP model on local communities, for example in terms of its effects on mental health literacy and stigma. Work is also needed to estimate the economic impact of the AMP model (in isolation or embedded in wider health strategies) in terms of both direct costs and benefits in health care use, and indirect societal costs and benefits.

Before the AMP model can be proposed as an effective method for increasing equity of access to high quality primary mental health care, it should be tested at scale and in other settings. Mixed methods designs will continue to be needed, and larger studies will provide opportunity for statistical analysis of quantitative outcomes. Practice-level referral and recruitment numbers could be analysed using regression models with appropriate adjustment of standard errors for the 'clustering' of practices within localities. Interactions between the different interventions could be explored in such models: characteristics of the practice population could also be considered. It will also be important to consider the extent to which future studies retain fidelity to the core features of our model [35].

Our focus has been on under-served groups in urban settings within a country with a strong infrastructure of primary care services and a well-developed range of existing psychological therapies. The model might operate less - or perhaps more - effectively in countries where primary care structures are not firmly established [36], or where there is limited access to conventional psychological therapies. It is not easy to maintain focus on the mental health needs of marginalized groups, even within affluent societies. Voluntary community-based organisations, crucial to the success of our community engagement strategies, are vulnerable to change in economic, cultural and political circumstances [23, 37].

## Conclusions

While we consider our findings to be widely applicable, it is essential to remain mindful of their contextual nature [34]. The three elements of the AMP model will have the same general structure if applied in other localities, or with focus on other under-served groups, but the details of their content and processes, and hence their anticipated outcomes, will be different. This will be even more the case for those planning to re-design primary mental health care services and improve access for under-served groups elsewhere in the world, with radically different health care systems. Nevertheless, our findings may be instructive to public health workers, family doctors and others wishing to enhance access to high quality mental health care for under-served groups.

## Abbreviations

AMP: Improving **A**ccess to **M**ental Health in **P**rimary Care
GP: General (medical) practitioner
